# Mutation analysis of 272 Spanish families affected by autosomal recessive retinitis pigmentosa using a genotyping microarray

**Published:** 2010-12-03

**Authors:** Almudena Ávila-Fernández, Diego Cantalapiedra, Elena Aller, Elena Vallespín, Jana Aguirre-Lambán, Fiona Blanco-Kelly, M. Corton, Rosa Riveiro-Álvarez, Rando Allikmets, María José Trujillo-Tiebas, José M. Millán, Frans P.M. Cremers, Carmen Ayuso

**Affiliations:** 1 Genetics Department, IIS-Fundación Jiménez Díaz, Madrid, Spain; 2 Centre for Biomedical Research on Rare Diseases (CIBERER), Spain; 3 Fundación para la Investigación, Hospital Universitario La Fe, Valencia, Spain; 4 Department of Ophthalmology, Columbia University, New York, NY; 5 Department of Pathology and Cell Biology, Columbia University, New York, NY; 6 Department of Human Genetics, Radboud University Nijmegen Medical Centre, Nijmegen, The Netherlands; 7 Nijmegen Centre for Molecular Life Sciences, Radboud University Nijmegen Medical Centre, Nijmegen, The Netherlands

## Abstract

**Purpose:**

Retinitis pigmentosa (RP) is a genetically heterogeneous disorder characterized by progressive loss of vision. The aim of this study was to identify the causative mutations in 272 Spanish families using a genotyping microarray.

**Methods:**

272 unrelated Spanish families, 107 with autosomal recessive RP (arRP) and 165 with sporadic RP (sRP), were studied using the APEX genotyping microarray. The families were also classified by clinical criteria: 86 juveniles and 186 typical RP families. Haplotype and sequence analysis were performed to identify the second mutated allele.

**Results:**

At least one-gene variant was found in 14% and 16% of the juvenile and typical RP groups respectively. Further study identified four new mutations, providing both causative changes in 11% of the families. Retinol Dehydrogenase 12 (*RDH12*) was the most frequently mutated gene in the juvenile RP group, and Usher Syndrome 2A (USH2A) and Ceramide Kinase-Like (*CERKL*) were the most frequently mutated genes in the typical RP group. The only variant found in *CERKL* was p.Arg257Stop, the most frequent mutation.

**Conclusions:**

The genotyping microarray combined with segregation and sequence analysis allowed us to identify the causative mutations in 11% of the families. Due to the low number of characterized families, this approach should be used in tandem with other techniques.

## Introduction

Retinitis pigmentosa (RP, OMIM 268000) is an inherited retinal dystrophy caused by a progressive loss of photoreceptors. Typically, the first symptom of the disease is night blindness, which is followed by a loss of peripheral vision and, in most cases, cone degeneration in the late stage. Its prevalence is approximately 1/4000 worldwide [1]. RP may be transmitted in all inheritance patterns. In addition, sporadic cases (sRP) have been described, representing 40%–50% of non-syndromic RP cases [1]. To date, 49 genes have been associated with RP, 32 of which are associated with autosomal recessive retinitis pigmentosa (see arRP at RetNet). However, only a little more than 50% of RP cases can be explained by mutations in these genes [2]. Due to arRP’s phenotypic and genetic heterogeneity, its molecular diagnosis is highly complex and time-consuming.

Currently, different genotyping techniques, such as single-strand conformation analysis [3], denaturing high-performance liquid chromatography (HPLC) [4], arrayed primer extension (APEX) analysis [5], and resequencing microarrays [6], are employed for the detection of mutations associated with disorders showing high genetic and allelic heterogeneity.

Several APEX arrays (Asper Biotech Ltd.; Tartu, Estonia) have been designed for syndromic and non-syndromic retinal dystrophies (e.g., Leber congenital amaurosis, Stargardt disease, Usher syndrome, Bardet-Biedl syndrome, and autosomal recessive and autosomal dominant retinitis pigmentosa) to identify the genetic cause of the disease.

The aim of this work was to identify the causative mutations in a panel of Spanish subjects affected by autosomal recessive RP (arRP) or sporadic juvenile RP and typical RP. A complete and efficient characterization of these patients would allow each patient to receive a more accurate prognosis and affected families to receive appropriate genetic counseling. Additionally, these individuals might benefit from upcoming therapeutic methods.

We studied a cohort of 272 unrelated Spanish families affected by autosomal recessive or sporadic juvenile RP, and typical RP. All cases were tested using the arRP-specific APEX genotyping microarray, followed by haplotype and sequence analysis.

## Methods

### Patients

A total of 272 unrelated Spanish families affected by autosomal recessive and sporadic non-syndromic retinal dystrophy were studied. Informed consent was obtained from all individuals recruited in accordance to the tenets of the Declaration of Helsinki (Seoul, 2008). Two different groups of patients—86 families with juvenile RP and 186 families with typical RP (onset after the age of 10)—were formed and studied independently, according to their clinical ophthalmic diagnosis. Juvenile RP was the classification for patients who complained of night blindness and visual field loss before the age of 10 years. These families were also classified based on the inheritance pattern (according to the modified criteria published by Ayuso et al. [7], which considers sRP plus consanguinity to be arRP: 107 families with arRP and 165 families with sRP.

In addition, 50 randomly selected DNA samples (100 chromosomes each) were taken from a healthy Spanish control population and analyzed to establish the prevalence of the new mutations identified in this study.

### Procedures

DNA was extracted from peripheral blood leukocytes collected in EDTA tubes using an automated DNA extractor (BioRobot EZ1; Qiagen; Hilden, Germany).

Mutational screening was performed, of one affected member of each family, using a genotyping microarray based on APEX technology. An APEX reaction is a genotyping method based on a single base extension, in which hundreds to thousands of variations in the genome are simultaneously analyzed in a single multiplexed reaction. This approach ensures highly specific discrimination without allele-specific hybridization, because the primer to be extended anneals just adjacent to the DNA base that needs to be identified. The complete description of this methodology can be found at the AsperBio website, and has been previously published elsewhere [8]. The chip included all known mutations from the coding region and adjacent intronic sequences of arRP genes. At the start of this study, in 2006, the chip included a total of 501 variants in 16 genes: Ceramide Kinase-Like (*CERKL*), Rod cGMP-gated Channel Alpha Subunit (*CNGA1*), Rod cGMP-gated Channel Beta Subunit (*CNGB1*), c-Mer Proto-oncogene Tyrosine Kinase (*MERKT*), cGMP Phosphodiesterase Alpha Subunit (*PDE6A*), Rod cGMP Phosphodiesterase Beta Subunit (*PDE6B*), Nuclear Receptor Transcription Factors (*PNR*), Retinol Dehydrogenase 12 (*RDH12*), RPE-retinal G protein-coupled Receptor (*RGR*), Retinaldehyde-Binding Protein 1 (*RLBP1*), Arrestin (s-antigen) *(SAG),* Tubby Like Protein 1 *(TULP1),* Crumbs Homolog 1 *(CRB1),* Retinal Pigment Epithelium-specific Protein 65 kDa *(RPE65),* Usher Syndrome 2A *(USH2A),* and Clarin 1 *(USH3A)*.

All detected variants were confirmed by sequence analysis. The sequence reaction was performed with a Big-dye DNA Sequencing Kit (version 3.1; Applied Biosystems; Foster City, CA). Sequence products were resolved in an ABIPrism 3130 (Applied Biosystems).

Haplotype analysis studies were performed using microsatellite markers, located within a determined interval of the candidate gene, in those arRP families in which the microarray detected one mutated allele. The markers were chosen from the literature: *CNGA1* from Zhang et al. [9] and Kondo et al. [10], *SAG,* and *USH2A* from Kondo et al. [10], *CRB1* from Vallespín et al. [11], and *PDE6A* from Chavanás et al. [12]. Upon detection of cosegregation in the family, we performed bidirectional sequence analysis of the exons and flanking intronic regions to identify the second mutated allele. To determine the parental origin in the cases in which the microarray detected two mutated alleles, cosegregation analysis of the variants was performed by sequence analysis.

Novel sequence variants found were tested for their presence in healthy control individuals by restriction fragment length polymorphism analysis of BanI for the *RDH12* c.278T>C (p.Leu93Pro) gene variant, and by sequence analysis for the *RPE65* c.457A>G (p.Thr153Ala), *USH2A* c.3713C>G (p.Thr1238Arg) and for the previously described variant: c.12575G>A (p.Arg4192His).

Sorting intolerant from tolerant (SIFT) analysis was used to predict the potential impact of the variants found in this study. A SIFT score below 0.05 is predicted to be pathogenic, while SIFT scores above 0.05 are considered tolerated.

## Results

### Genotyping microarray analysis of juvenile RP versus typical RP

The genotyping microarray was used for diagnosis. Accordingly, polymorphisms were excluded before the analysis. At least one mutation was found in 12 out of 86 (14%) families with juvenile RP and in 30 out of 186 (16%) typical RP families studied. Of all RP alleles studied, 18 out of 172 (10.5%) juvenile RP alleles and 46 out of 372 (12%) typical RP alleles were identified as sequence variants.

The different variants detected with the genotyping microarray and confirmed by sequence analysis in both groups of patients are shown in Table 1 and Table 2. Two false positives were detected in the juvenile RP group (RP-0337 and RP-1015; data not shown).

**Table 1:** Mutations identified in patients with juvenile RP.

**Juvenile RP**						
			**Mutation 1**		**Mutation 2**	
**Family**	**Inheritance pattern**	**Gene**	**Nucleotide change**	**Protein defect**	**Nucleotide change**	**Protein defect**
RP-0531	ARRP	*CNGA1*	c.94G>A	p.Arg32Stop	c.94G>A	p.Arg32Stop
RP-0561	ARRP	*CRB1*	c.2234C>T	p.Thr745Met	c.3988G>T	p.Glu1330Stop^
RP-1311	SRP	*CRB1*	c.611_617delAAATAGG	p.Ile205AspfsX13		
RP-0235	ARRP	*PDE6A*	c.304C>A	p.Arg102Ser		
RP-0341	ARRP	*PDE6A*	c.998+1G>A	Splicing defect	c.1705C>A	p.Gly569Lys^
RP-0054	SRP	*PDE6B*	c.810C>A	p.Cys270Stop	c.810C>A	p.Cys270Stop
RP-0340	ARRP	*RDH12*	c.464C>T	p.Tyr155Ile	c.464C>T	p.Tyr155Ile
RP-0379	SRP	*RDH12*	c.375T>A	p.Asn125Lys	c.701G>A	p.Arg234Hys
RP-1339	SRP	*RDH12*	c.295C>A	p.Leu99Ile	**c.278T>C**	**p.Leu93Pro^**
RP-0979	ARRP	*RLBP1*	c.451C>T	p.Arg151Gln	c.451C>T	p.Arg151Gln
RP-1115	ARRP*	*RPE65*	c.95–2A>T	Splicing defect	**c.457A>G**	**p.Thr153Ala^**
RP-1206	SRP	*SAG*	c.577C>T	p.Arg193Stop	c.577C>T	p.Arg193Stop

**^**Variants detected by sequence analysis (novel variants are in bold). Consanguineous family: * parents first cousins.

**Table 2:** Mutations identified in patients with typical RP.

**Typical RP**						
			**Mutation 1**		**Mutation 2**	
**Family**	**Inheritance pattern**	**Gene**	**Nucleotide change**	**Protein defect**	**Nucleotide change**	**Protein defect**
RP-0211#	SRP	*CERKL*	c.769C>T	p.Arg257Stop	c.769C>T	p.Arg257Stop
RP-0218#	SRP*	*CERKL*	c.769C>T	p.Arg257Stop	c.769C>T	p.Arg257Stop
RP-0320#	ARRP*	*CERKL*	c.769C>T	p.Arg257Stop	c.769C>T	p.Arg257Stop
RP-0325#	SRP	*CERKL*	c.769C>T	p.Arg257Stop	c.769C>T	p.Arg257Stop
RP-0535#	ARRP	*CERKL*	c.769C>T	p.Arg257Stop	c.769C>T	p.Arg257Stop
RP-0595#	ARRP**	*CERKL*	c.769C>T	p.Arg257Stop	c.769C>T	p.Arg257Stop
RP-0657	SRP	*CERKL*	c.769C>T	p.Arg257Stop	c.769C>T	p.Arg257Stop
RP-0828#	SRP**	*CERKL*	c.769C>T	p.Arg257Stop	c.769C>T	p.Arg257Stop
RP-1159	ARRP	*CERKL*	c.769C>T	p.Arg257Stop	c.769C>T	p.Arg257Stop
RP-0159	ARRP*	*CNGA1*	c.94G>A	p.Arg32Stop		
RP-1080	SRP	*CNGA1*	c.94G>A	p.Arg32Stop	c.94G>A	p.Arg32Stop
RP-1147	ARRP	*CNGA1*	c.94G>A	p.Arg32Stop		
RP-1106	SRP	*CRB1*	c.2681A>G	p.Asn894Ser		
RP-0881	SRP	*PDE6A*	c.305G>A	p.Arg102His	c.305G>A	p.Arg102His
RP-1023	SRP	*SAG*	c.577C>T	p.Arg193Stop		
RP-1292	ARRP	*SAG*	c.577C>T	p.Arg193Stop		
RP-0134	SRP	*USH2A*	c.1606T>C	p.Cys536Arg		
RP-0204	ARRP	*USH2A*	c.2276G>T	p.Cys759Phe	c.9799T>C	p.Cys3267Arg
RP-0260	SRP	*USH2A*	c.9799T>C	p.Cys3267Arg	c.10073G>A	p.Cys3358Tyr^
RP-0332	ARRP	*USH2A*	c.2276G>T	p.Cys759Phe	c.2276G>T	p.Cys759Phe
RP-0404	SRP**	*USH2A*	c.2167+5G>A	Splicing defect	c.2167+5G>A	Splicing defect
RP-0467	ARRP	*USH2A*	c.2276G>T	p.Cys759Phe		
RP-0653	SRP	*USH2A*	c.2276G>T	p.Cys759Phe	c.12575G>A	p.Arg4192His^
RP-0721	SRP	*USH2A*	c.2276G>T	p.Cys759Phe	**c. 3713C>G**	**p.Thr1238Arg^**
RP-0849	ARRP*	*USH2A*	c.2276G>T	p.Cys759Phe	c.2276G>T	p.Cys759Phe
RP-0930	SRP	*USH2A*	c.2276G>T	p.Cys759Phe	c.2276G>T	p.Cys759Phe
RP-1016	ARRP	*USH2A*	c.2276G>T	p.Cys759Phe		
RP-1053	SRP	*USH2A*	c.2276G>T	p.Cys759Phe	**c.13745del**	**p.Ile4582Lysfs14^**
RP-1059	SRP	*USH2A*	c.2276G>T	p.Cys759Phe		

**^**Variants detected by sequence analysis (novel variants are in bold). Consanguineous families: * parents first cousins ** parents second cousins. # These data were published before as a common phenotype [19].

### Segregation analysis of the families in which one variant was found by arrayed primer extension analysis

**Figure 1:**
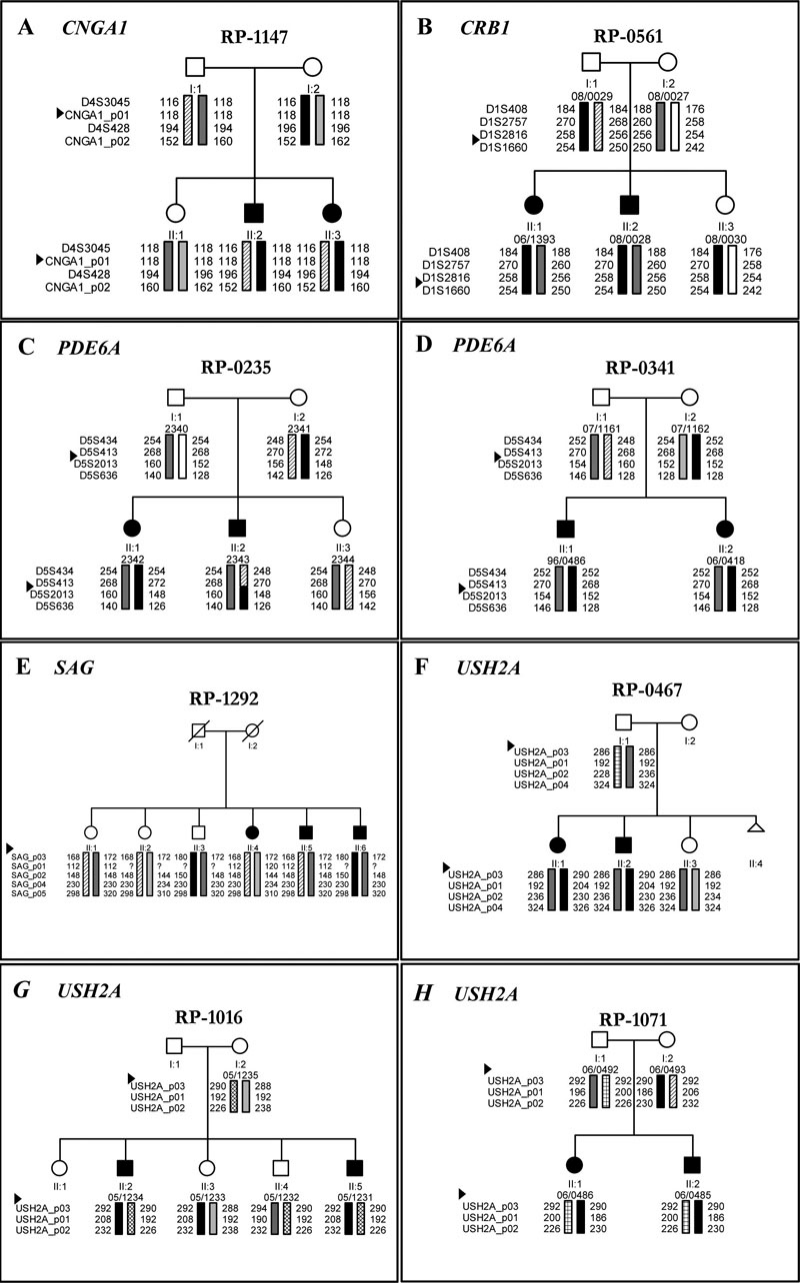
Haplotype analysis of the chromosome segments encompassing arRP genes in those arRP families (**A**-**H**) in which the microarray detected one mutated allele. For the RP-1292 (**E**) the haplotype analysis shows the studied gene does not co-segregate with the disease. ►Where the gene is located.

**Figure 2:**
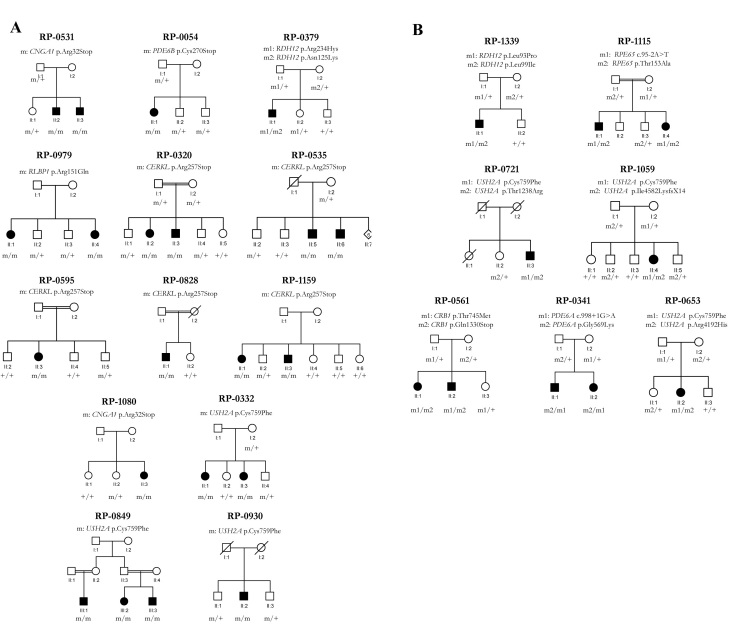
Family pedigrees in which the co-segregation of the detected mutations was performed. “+” wild type allele, “m, m1 and m2” mutated alleles. **A**: Pedigrees of families with RP mutations. **B**: Pedigrees of families with RP mutations.

Figure 1 shows the results of the cosegregation analysis by microsatellite markers in those arRP families in which the microarray detected one mutated allele and other family members were available. In the RP-1147, RP-0561, RP-0341, RP-0467, RP-1016, and RP-1071 families, the studied gene variants cosegregated with the disease, while those for RP-1292 did not co-segregate. For RP-0235, the segregation analysis for *PDE6A* showed a recombination between the D5S413 and D5S2013 markers. For RP-0159, the p.Arg32Stop mutation in CNGA1 is not the causative mutation of the disease in this family since this mutation does not segregate with the disease (data not shown). Figure 2A shows the results of the segregation analysis of those families with two mutations identified by the microarray when other family members were available.

### Sequence analysis

For the families with one mutated allele, one patient from each arRP family was sequenced for the gene (*CNGA1*, *CRB1*, *PDE6A,* or *USH2A*) that cosegregated with the disease. In the sporadic cases, the individuals who presented one mutated allele were also subjected to sequence analysis of the gene. The index case of the RP-0134 family could not be studied due to poor sample quality.

Using sequence analysis, we identified four novel and four previously described variants (Table 1 and Table 2). All novel pathogenic missense variants and the previously described variant p.Arg4192His in *USH2A* were tested in a healthy Spanish control population. None out of 100 chromosomes tested showed these changes. SIFT analysis for the novel substitutions predicted these changes would affect the protein function (SIFT score<0.05), except for the p.Thr153Ala mutation in *RPE65* (the SIFT analysis predicted this change would be tolerated). The cosegregation of the mutations with the disease was performed when other family members were available. Family pedigrees are shown in Figure 2B.

Three out of four previously described variants were not included in the microarray analysis at the time of the study: p.Glu1330Stop in *CRB1* [13], p.Cys3358Tyr, and p. Arg4192His in *USH2A* [14] (detected by sequence analysis in RP-0561, RP-0260, and RP-0653, respectively). For RP-0341, the second mutated allele, p.Gly569Lys in *PDE6A*, was found by sequence analysis. That change was included in the genotyping microarray, so in that case, the result was considered a false negative. For families RP-1147, RP-1311, RP-1106, RP-1023, RP-0467, RP-1016, RP-1053, and RP-1071, which presented one mutated allele found by the genotyping microarray, the screening of the respective genes did not show any other pathogenic variants.

## Discussion

Because of the high genetic heterogeneity of arRP, to identify the genetic cause in these patients is expensive and time-consuming. In this study, 272 Spanish families affected by arRP were analyzed by a genotyping microarray, followed by sequence analysis of the candidate genes to establish a fast and effective genetic diagnosis. With this approach, we were able to identify two pathologic variants in 30 (11%) families one variant in 12 (4.5%) families.

In the juvenile RP group, there was not a frequent mutation. However, for the typical RP patients, the most frequent mutation was p.Arg257Stop in *CERKL*, followed by the p.Cys759Phe mutation in *USH2A*.

Among the typical RP families, *USH2A* was the most frequently mutated gene, with 23 out of 372 alleles. *USH2A* has been shown to be involved in Usher syndrome [15] and in RP without hearing loss [16]. In our cohort of patients, *USH2A* accounted for 7% (14 out of 186) of typical RP cases—a frequency similar to the one found in other populations [17]. The p.Cys759Phe variant was the second-most-frequent mutation, accounting for 3.8% (14 out of 372 alleles) of typical RP families; this percentage was higher than reported elsewhere [18]. *CERKL* was the second most-mutated gene in Spanish patients affected by typical RP. The p.Arg257Stop mutation is the only one found in the Spanish arRP population to date. In our cohort of patients, the p.Arg257Stop mutation in the *CERKL* gene accounted for 4.8% (9 out of 186) of typical RP cases. In all these cases, the mutation was found homozygously. The percentage found in this study was higher than the one described by us previously [19], due to the juvenile RP families having not been included in this percentage. In addition, despite the wide geographic distribution of these families, a common ancestry was described [13], as all the affected members of the seven families studied shared the same haplotype.

Among the juvenile RP families, *RDH12* was the most frequently mutated gene, with six out twenty mutated alleles. Mutations in *RDH12* have been associated with early-onset autosomal recessive retinitis pigmentosa [20]. In our cohort of patients, *RDH12* accounted for 3.4% (3 out of 86) of the families. This frequency is similar to the one reported in a previous study done on a Spanish population [21], though higher than the frequency (2.2%) found in a population from the United States [20]. It follows from this result that the Spanish population affected by retinal dystrophies presents different frequencies for some genes, compared to other populations [11].

There were no differences when the results were compared between arRP and sRP families. Therefore, as has been described before [22], our results support the conclusion that a fair majority of the sporadic cases, which accounts for 40%–50% of non-syndromic RP cases, present an autosomal recessive inheritance.

Novel variants identified by sequencing analysis

We identified four novel pathogenic variants in three different genes. In the *USH2A* gene, we found the p.Ile4582LysfsX14 and p.Thr1238Arg mutations. In the *RDH12* gene, we identified the p.Leu93Pro variation. The predicted SIFT score (<0.05) and the absence of these variants in healthy controls help establish the pathogenicity of these variants. The SIFT program predicted that the missense variant p.Thr153Ala in *RPE65* would be tolerated (SIFT score >0.05).

However, the absence of change in control population and the cosegregation with the disease in the family argues in favor of its pathogenicity.

After the screening of *USH2A* for RP-0653 the p.Arg4192His change, a previously benign variant, was detected. However, the absence in healthy controls and the cosegregation of the change with the family, argue in favor of this variant being related to the retinal dystrophy in the family. Although it is reasonable to support this conclusion, additional studies should be performed to elucidate the pathogenic role of this variant.

In several families (RP-1311, RP-1147, RP-1106, RP-1023, RP-0467, RP-1016, RP-1053, and RP-1071), the second mutated allele could not be determined. The absence of a second pathogenic mutation in these genes could be explained in different ways. The pathogenic mutations could lie in other genes that interact with *CRB1*, *CNGA1*, *SAG*, or *USH2A*, as previously described among some families with RP [23]. It is also possible that the second mutation could not be detected by sequence analysis because of the limitations of the technique; large deletions have been described in *USH2A* as a cause of Usher syndrome [24]. In addition, the undetected *USH2A* mutations in these cases might be located within the promoter region, intronic sequences, and 3′ and 5′ untranslated regions (UTR). Another important consideration is why only one variant was found in some families, including RP-0235, RP-0159, and RP-1292. In none of these did the detected variants found by the APEX genotyping microarray cosegregate with the disease, despite the fact that these changes have been described as causative mutations in different studies. The reason is that there is a considerable mutational load in the general population. Rivolta et al. [25], assuming a total of 67 arRP genes making equal contributions, estimated that 10% of healthy individuals carries an arRP variant. Thus, some of the 12 out of 272 probands in which one pathologic variant was found may have been chance findings that were not related to retinal dystrophy.

The APEX technology provides a quick analysis of a large number of mutations at the same time, and allows new mutations to be added to the microarray analysis. However, it has some limitations. All detected changes have to be confirmed by sequence analysis. The array incorporates changes of an unknown pathologic nature. The most important disadvantage is that this microarray only tests previously reported mutations in known genes. Finally, this array does not include some recently identified RP genes such as the Eyes Shut homolog *(EYS)* gene, which is presumed to be a major gene for recessive RP in the Spanish population [26].

The use of the genotyping microarray, combined with segregation and sequencing analysis, allowed us to identify the causative mutations in at least 11% of our cohort of patients, lower than that described using other disease-specific microarrays (such as the LCA [6] and Usher [11] genotyping microarray by Asper Biotech) in the Spanish population. This approach should be used in tandem with other approaches such as exome sequencing and indirect methods (whole-genome single-nucleotide polymorphism [SNP] genotyping combined with linkage analysis and homozygosity mapping). This strategy would allow us to identify new mutations and loci. A complete and efficient characterization of these patients enables them to receive appropriate genetic counseling and to contribute to the development of gene-based therapyfor themselves and others.
